# Medical comorbidities in children and adolescents with autism spectrum disorders and attention deficit hyperactivity disorders: a systematic review

**DOI:** 10.1007/s00787-017-1020-0

**Published:** 2017-07-03

**Authors:** Jet B. Muskens, Fleur P. Velders, Wouter G. Staal

**Affiliations:** 1 Karakter Child and Adolescent Psychiatry University Center Nijmegen, Reinier Postlaan 12, Nijmegen, The Netherlands; 2 Department of Psychiatry, Radboud University Medical Center, Nijmegen, The Netherlands; 3 Department of Psychiatry, Brain Center Rudolf Magnus, University Medical Center, Utrecht, The Netherlands; 4 Department of Cognitive Neuroscience, Donders Institute for Brain, Cognition and Behaviour Radboudumc, Nijmegen, The Netherlands

**Keywords:** Child and adolescent psychiatry, Medical disorders, Comorbidity, Developmental disorders, Autism spectrum disorder, Attention deficit hyperactivity disorder

## Abstract

Somatic disorders occur more often in adult psychiatric patients than in the general adult population. However, in child and adolescent psychiatry this association is unclear, mainly due to a lack of integration of existing data. To address this issue, we here present a systematic review on medical comorbidity in the two major developmental disorders autism spectrum disorder (ASD) and attention deficit hyperactivity disorder (ADHD) and formulate clinical recommendations. The literature was searched using the PubMed and PsycINFO databases (2000–1 May 2016) with the keywords “[((child and adolescent) AND (Autism OR Attention Deficit Hyperactivity Disorder* OR ADHD)) AND (“Cardiovascular Diseases” [Mesh] OR “Endocrine System Diseases” [Mesh] OR “Immune System Diseases” [Mesh] OR “Neurobehavioral Manifestations” [Mesh] OR “Gastrointestinal Disorders” [Mesh] OR Somatic OR Autoimmune disease OR Nervous system disease OR Infection OR Infectious disease)]. Two raters independently assessed the quality of the eligible studies. The initial search identified 5278 articles. Based on inclusion and exclusion criteria 104 papers were selected and subsequently subjected to a quality control. This quality was assessed according to a standardized and validated set of criteria and yielded 29 studies for inclusion. This thorough literature search provides an overview of relevant articles on medical comorbidity in ADHD and/or ASD, and shows that medical disorders in these children and adolescents appear to be widespread. Those who work with children with ASD and/or ADHD should be well aware of this and actively promote routine medical assessment. Additionally, case–control studies and population-based studies are needed to provide reliable prevalence estimates. Future studies should furthermore focus on a broader evaluation of medical disorders in children and adolescents with ADHD and/or ASD to improve treatment algorithm in this vulnerable group.

## Introduction

Medical disorders occur more often in adult psychiatric patients than in the general population [1]. Moreover, somatic symptoms may cause or enhance psychiatric symptoms [2–4]. A comprehensive review of comorbidity of mental and medical disorders in adults was presented by The Robert Wood Johnson Foundation in 2011, which included its the prevalence, origins and models for effective treatment [5]. More than 68% of adults with a mental disorder had at least one medical condition. Thereby, comorbidity was associated with elevated symptom burden, functional impairment, decreased length and quality of life and increased costs. The pathways between psychiatric symptoms and medical disorders are mostly, if known, complex and bidirectional. For example, adults with hypothyroidism are at risk for mood disorders [6, 7], and patients with schizophrenia and bipolar disorder have an increased risk of developing metabolic syndrome, due to their psychiatric disease as well as the use of antipsychotic drugs [8, 9]. Moreover, adults with mental health problems are more likely to have sedentary lifestyles and poor diets [5]. Different studies showed that patients with schizophrenia, bipolar disorder, or major depression, report less physical activity compared with those without mental disorders, and tend to eat foods that are high in fat and calories while avoiding fruits and vegetables [10, 11].

The increased risk for mental health problems has also been found in children with medical disorders. Findings of the Isle of Wight studies of Sir Michael Rutter and colleagues [12] starting in 1964–1965, already reported that 12% of children with somatic disorders (non-neurological) exhibited mental health problems compared to 7% of children in the general population [13, 14]. Also, significantly higher rates of mental health problems were reported in children with epilepsy: 29% in children with uncomplicated epilepsy and 58% in those with complicated epilepsy (i.e., structural brain abnormalities and seizures).

Several systematic reviews have been published on the association between specific medical disorders and child psychiatric disorders, for instance on the association between epilepsy and psychopathology [15], atopic diseases and mental health [16, 17], inflammation and neuropsychiatry [18], headaches/migraine and psychopathology [19, 20], HIV/AIDS and mental health [21], gastro-intestinal dysfunction and ASD [22], immune factors and ASD [23], phenylketonuria and ADHD [24]. Aldinger and colleagues aimed to identify clustering of medical disorders in children with autism. In this study, the prevalence of medical conditions ranged from 10.7 to 77.4%. A co-occurrence of sleeping problems and gastro-intestinal disturbances in children with autism was found. Also, the co-occurrence of gastro-intestinal disturbances, seizures and sleep problems predicted more severe behavioral symptoms in children with ASD [25]. Buie et al. concluded that medical disorders, such as gastro-intestinal problems, occur commonly in individuals with ASD [26]. However, medical conditions may remain undiagnosed in children with ASD due to atypical presentation of symptoms. Nonverbal or minimally verbal ASD individuals cannot verbally express pain or discomfort and instead often demonstrate their level of discomfort through disruptive behaviors, including aggressions and self-injury [26]. Even patients with ASD who acquire verbal communication skills may have difficulty describing subjective experiences or symptoms. Clinicians should be aware that problem behavior in ASD may be the primary or sole symptom of the underlying medical disorder. Even more so, Schieve and colleagues showed that children with specific developmental delays (autism, ADHD, learning disorder and intellectual disability) were more likely to have certain medical conditions than children without these delays [27]. Based on this finding, Schieve and colleagues state that children with developmental delays require increased pediatric health services and specialist services, both for their core functional deficits as for their health problems [27]. However, these results were based on parents or caregivers report, without clinical confirmation on developmental delays or medical conditions.

Following the data from reviews and clinical studies, Merikangas and colleagues performed a study on comorbidity of mental and physical conditions and functional impairments in 9014 children and adolescents [28]. In particular, a strong association between ADHD and neurologic disorders (seizures and epilepsy) was found. This association between neurologic disorders that affect brain systems and behavioral disorders is in line with other studies examining developmental disorders such as autism, ADHD, and neurobehavioral problems [29, 30]. It may implicate dysfunction in the underlying network and common genetic and/or environmental risk factors [31–34].

In a previous pilot study we evaluated the outcome of medical screening at referral in children and adolescents with different psychiatric disorders. This screening revealed new somatic findings in 56% of the subjects [35]. These findings included a broad spectrum of medical concerns, including weight and length problems, high levels of thyroid hormone, dyslipidemia, anemia, vitamin D and vitamin B12 deficiency and dysmorphic anomalies. Some of these results required consultation from other medical specialists, whereas others had direct implications for daily medical practice, such as adjustments in psychopharmacologic treatment and/or participation in prevention programs for overweight.

These findings all contribute to the increasing awareness about the association between somatic and mental symptoms in developmental disorders and may suggest potential mechanisms such as common genetic pathways [28]. Also, the simultaneous assessment of medical and psychiatric disorders could be of major value. It seems important that the interpretation of meaningful somatic findings is done by clinicians who are able to relate these findings to differential diagnostic considerations for both medical and psychiatric interventions.

Most of the available literature on medical disorders in child and adolescent psychiatric disorders relates to ASD and ADHD, but there is a lack of integration of existing data, hampering the interpretation of findings. Therefore, this systematic review focuses on the association of medical disorders with autism and/or ADHD in children and adolescents and provides recommendations for future studies. Our hypothesis is that children with ADHD and ASD are at increased risk for a broad spectrum of medical disorders.

## Methods

### Search criteria

The literature was searched using the PubMed and PsycINFO databases (January 2016) with the following keywords: [((child and adolescent) AND (Autism OR Attention Deficit Hyperactivity Disorder* OR ADHD)) AND (“Cardiovascular Diseases” [Mesh] OR “Endocrine System Diseases” [Mesh] OR “Immune System Diseases” [Mesh] OR “Neurobehavioral Manifestations” [Mesh] OR “Gastrointestinal Disorders” [Mesh] OR Somatic OR Autoimmune disease OR Nervous system disease OR Infection OR Infectious disease)]. To be included in the present review, the articles had to be written in English language and published between 2000 and the first of May 2016.

### Screening procedure

First, the papers were screened on title and abstracts, which was conducted independently by two of the authors (JM and FV) in order to assess the eligibility of the studies. Second, this selection of papers was screened based on the full text. The third author (WS) was consulted in case of differences in scoring by the other two authors. The inclusion and exclusion criteria were as follows.

Inclusion criteria:

1. Sample subjects had to fulfill criteria for autism spectrum disorders (ASD) or attention deficit hyperactivity disorders (ADHD) by DSM-IV [36], DSM-IV-R [37], DSM 5 [38] or ICD-10 [39] or scores above a clinical threshold obtained using instruments for the assessment of ADHD or ASD. ASD encompasses autistic disorders, Asperger’s disorder and pervasive developmental disorder-not otherwise specified (PDD-NOS) within the DSM classification system, and childhood autism, infantile autism, atypical autism and Asperger’s syndrome in the ICD classification system. Considering the diagnosis of ADHD, studies on attention deficit disorder with or without hyperactivity were both included.
2. A medical or somatic disorder diagnosed by a clinician, from National Health Surveys or databases, or parents’ report.
3. The age of the study participants was 18 years or younger including population-based studies started during childhood with follow-up >18 years.

Exclusion criteria:

1. Study design: case report, case series, letter to the editor, editorial, or data solely presented by means of a poster presentation.
2. Study focus: intervention studies in which subjects were selected for pharmacological treatment of ADHD, exposure on chemicals, effects of dietary supplementation, perinatal complications and studies including mental retardation and/or syndromes.

Third, the quality of each study was assessed according to a standardized and validated set of criteria based on the protocols of the Cochrane Database of Systematic Reviews [40]. The papers were scored independently on the 6 criteria below by 2 raters (JM and FV), in order to quantify the methodologic aspects and the quality standard of each study. Each study could obtain a maximum of 6 points (1 point per criterion). Papers with a quality assessment score of 5 or higher were considered of high quality and selected for this review. Inter-rater disagreement was primarily solved by discussion or arbitration of a third rater (WS) to enhance uniformity in the scoring procedure. The following six criteria were used:

1. Comparison group(s): the presence of at least 1 comparison group (control group), preferably a sample of healthy children from the same population or region as the children with medical disorders or ASD/ADHD.
2. Sample selection: a random selection strategy should be used.
3. Design: a case–control design based on quantitative information or population-based cohort studies.
4. Diagnostic validity: DSM criteria, ICD 10 by clinicians (not only by parent, teacher or self- reports).
5. Outcome measures: medical disorder or ASD/ADHD.
6. Statistical analyses: hypothesis testing using appropriate statistical analyses should be performed.

## Results

**Fig. 1:**
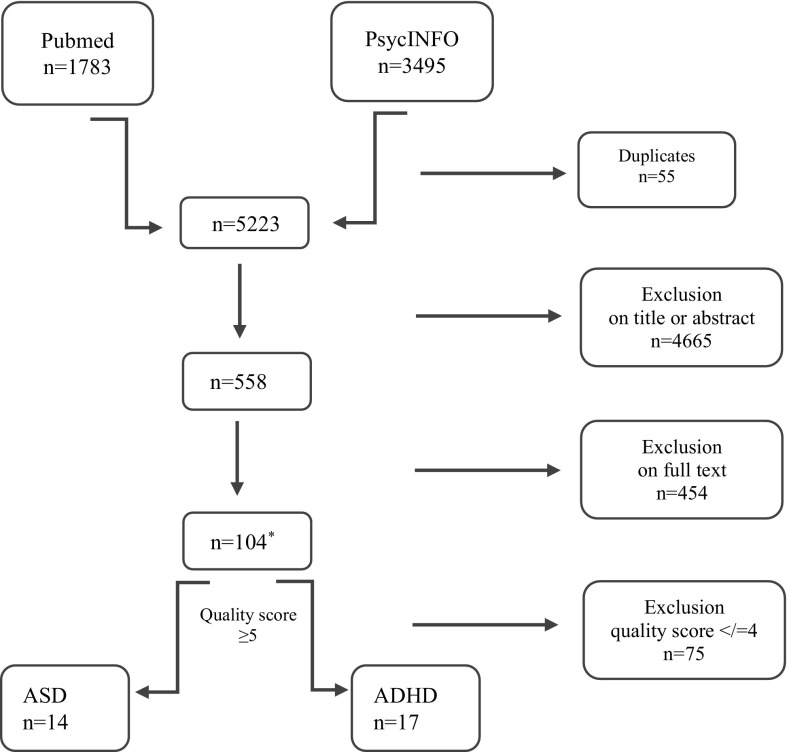
Flowchart of study search and inclusion in the review. *Asterisk* two papers are listed under both ASD and ADHD and consequently double represented in the numbers

The initial search yielded in total 5278 papers (1783 and 3495 hits, respectively, of which 55 duplicates). 4665 papers were excluded according to the title and/or the abstract and 451 were excluded based on the full text. The remaining 104 papers were scored of which 29 papers were considered of high quality (5 or 6 points) and presented in this review. Two of these 29 papers focused on both ADHD and ASD, and are therefore listed in both tables (see Fig. 1).

Table 1 presents the papers that studied the association between ASD and medical disorders: 5 papers on immunology, 6 papers on gastroenterology, 1 paper on neurology and 2 papers on other medical disorders (see Table 1). In Table 2, the papers are listed that studied ADHD and medical disorders: 12 papers on immunology, 1 paper on neurology and 4 papers on other medical disorders (see Table 2).

**Table 1:** Autism spectrum disorders and medical disorders

	Authors	Year	Study size cases/controls	Age range (years)	Study design	Medical disorder
Immunology	Jyonouchi et al.	2008	26/212	1–18	Cross-sectional	Atopy, asthma, allergy and immunodeficiency
	MH Chen et al.	2014	14,812/6994	1–14	Case–control	Asthma, atopic dermatitis, allergic rhinitis, or allergic conjunctivitis
	Gentile et al.	2014	54/46	3–9	Cross-sectional	Antibody levels of HSV 1 and HSV 2
	Puig–Alcatraz et al.	2015	35/34	4–13	Cross-sectional	Homocysteine levels
	Zerbo et al.	2015	5565/27,825	3–26	Case–control	Immune-mediated conditions
Gastroenterology	Valicenti et al.	2006	50/100	4–12	Cross-sectional	Gastro-intestinal symptoms
	Mouridsen et al.	2010	118/336	0–57	Case–control	Gastro-intestinal diseases
	Wang et al.	2011	589/163	1–18	Cross-sectional	Gastro-intestinal problems
	Chandler et al.	2013	132/163	10–14	Cross-sectional	Gastro-intestinal symptoms
	Mouridsen et al.	2013	89/258	3–65	Case–control	Gastro-intestinal diseases
	Von Gontard et al.	2015	40/43	5–17	Case–control	Gastro-intestinal symptoms
Neurology	Mouridsen et al.	2013	4180/general population	4–31	Cross-sectional	Epilepsy
Other medical disorders	Van Tongerloo et al.	2012	49/81	8–16	Case–control	Medical comorbidity and referrals by a general practitioner
	Butwicka et al.	2015	9262/468,036	1–30	Case–control	Hypospadias

**Table 2:** Attention deficit hyperactivity disorder and medical disorders

	Authors	Year	Study size (cases/controls)	Age range (years)	Study design	Medical disorder
Immunology	Gau et al.	2008	86/172	4–16	Case–control	Enterovirus 71 central nervous system infection
	Leslie et al.	2008	3650/18,114	5–10	Case–control	Streptococcal infection
	Sanchez et al.	2009	22/22	6–14	Cross-sectional	Anti-basal ganglia antibodies
	Schmitt et al.	2009	1436/1436	6–17	Cross-sectional	Atopic eczema
	Suwan et al.	2011	40/40	5–15	Cross-sectional	Allergic sensitization and rhinitis
	Tsai et al.	2011	60,438/116,112	0–17	Cross-sectional	Allergic rhinitis
	Siyu et al.	2012	48,457/178,093	0–17	Cross-sectional	Allergic diseases
	Chen et al.	2013	4302/21,510	5–15	Case–control	Diabetes type I and II
	Chen et al.	2013	5811/23,244	7–23	Cross-sectional	Allergic diseases
	Tsai et al.	2013	4692/18,768	1–18	Case–control	Atopic diseases
	Bekdas et al.	2014	60/30	6–12	Cross-sectional	IgG levels of viruses
	Chen et al.	2014	14,812/6994	1–14	Case–control	Asthma, atopic dermatitis, allergic rhinitis, or allergic conjunctivitis
Neurology	Merikangas et al.	2015	9014	8–21	Cross-sectional	Physical conditions*
Other disorders	Dillon et al.	2007	79/27	5–13	Case–control	Adenotonsillectomy
	DeMaso et al.	2014	139/61	14–17	Cross-sectional	D-transposition of the great arteries
	Silva et al.	2014	11,902/27,304	0–18	Cross-sectional	Hospitalizations and physical conditions
	Butwicka et al.	2015	9262/468,036	1–30	Case–control	Hypospadias

* Allergy/immunology, cardiology, endocrine/metabolism, ear/nose/throat, gastroenterology, hematology, nephrology, neurology, oncology, orthopedics, pediatrics, pulmonology/airways, surgery and urology

### ASD and immunology

With regard to a possible association between ASD and immunological disorders, most papers studied allergic disease or autoimmune disease. Jyonouchi et al. evaluated whether one could clinically distinguish a subset of children with ASD and immunological disorders from other ASD children based on the frequency of infections [41]. In this sample, no significant differences were found considering the prevalence of atopy, asthma, food allergy, primary immunodeficiency between children in the ASD test group and controls (ASD controls, non-ASD controls with food allergy, non-ASD controls with frequent infections and normal controls).

Chen et al. concluded, however, that the presence of any atopic disease in early childhood increased the risk of developing ASD (hazard ratio: (HR) 3.40) in later life [42]. Greater numbers of atopic comorbidities (4 comorbidities HR: 4.29) were significantly related to a greater risk of developing ADHD and ASD.

Gentile et al. found similar rates of contact with Herpes Simplex Virus 1 and Herpes Simplex Virus 2 between children with ASD and healthy controls [43].

The study of Puig-Alcatraz et al. found that the increase in homocysteine concentration, suggestive for disorders of the immune system, was significantly correlated with the severity of the deficit in communication skills in children with ASD, but was unrelated to deficit in socialization or repetitive/restricted behavior compared to typically developing children [44].

Contrary to the study of Jynouchi et al., Zerbo et al. reported that allergies and autoimmune diseases were diagnosed significantly more often among children with autism than among controls (allergy: 20.6 vs. 17.7%, crude odds ratio (OR) = 1.22, 95% confidence interval (CI) 1.13–1.31; autoimmune disease: 1 vs. 0.76%, OR = 1.36, 95% CI 1.01–1.83), and asthma was diagnosed significantly less often (13.7 vs. 15.9%; OR = 0.83, 95% CI 0.76–0.90) [45]. Furthermore, psoriasis was seen more often in cases than in controls (0.34 vs. 0.15%; OR = 2.35, 95% CI 1.36–4.08).

### ASD and gastroenterology

The study of Valicenti et al. found that children with ASD had a significantly higher rate of gastro-intestinal (GI) symptoms than children with either typical development or other developmental disabilities (history of GI symptoms 70 vs 28 vs 42%, abnormal stool pattern 18 vs 4 vs 2%, food selectivity 60 vs 22 vs 36%) [46]. There was no association between a family history of autoimmune disease and GI symptoms in children with ASD.

The longitudinal population-based study of Mouridsen et al. found no evidence that patients with infantile autism (IA) were more likely than controls to have defined gastro-intestinal diseases during a 30.3-year observation period (30.5% against 30.7%) [47]. Only diseases of oral cavity (dental problems) were significantly associated with IA (20.3 vs 1.2%, *p* value < 0.0001).

In the study of Wang et al., parents retrospectively reported significantly more GI problems in children with ASD (249/589; 42%) compared with their unaffected siblings (20/163; 12%) (*p* < 0.001) [43]. The 2 most common gastro-intestinal problems in children with ASD were constipation (20%) and chronic diarrhea (19%). Next, individuals with ASD were grouped into 3 autism severity groups (Full Autism, Almost Autism, and Spectrum) based on their Autism Diagnostic Interview-Revised and Autism Diagnostic Observation Scale scores. Increased autism symptom severity was associated with higher odds of gastro-intestinal problems (AOR for trend = 2.63, 95% CI: 1.56–4.45).

The increased prevalence of GI symptoms was also found by Chandler et al. In this study, 46.5% of children with ASD had at least one individual lifetime GI symptom compared with 21.8% of typically developing children and 29.2% of the children with special educational needs [48]. No association was found between GI symptoms and intellectual ability, ASD severity, ASD regression or a faddy diet (arbitrary and often unusual likes and dislikes about food).

The study of Mouridsen et al. compared prevalence rates of gastro-intestinal tract diseases (esophagus, stomach, duodenum, gall bladder, biliary tract, pancreas, liver, peritoneum, intestines) in patients with PDD-NOS. In this study, conducted with data from the Danish National Hospital Register, the difference in prevalence of children with autism (24.7% (22/88)) and at least one diagnosis of any disease of the gastro-intestinal tract was not statistically significant (control group 18.2% (47/258)) (*p* = 0.22; odds ratio = 1.5; 95% confidence interval = 0.8–2.6). Interestingly, only hernia was significantly associated with autism 11.2 vs 4.7% (*p* = 0.04; OR = 2.6; 95% CI = 1.08–6.2). There were no children with inflammatory GI diseases, such as Crohn’s disease or ulcerative colitis in the autism group. Hence, in this study people with autism had about the same frequency of gastric, intestinal and hepatic diseases as had controls [49].

The study of Von Gontard et al. showed increased rates of nocturnal enuresis (30.0 vs 0%) and daytime urinary incontinence (25.0 vs 4.7%) in children with ASD compared to controls. Furthermore, ASD children had more lower urinary tract symptoms (LUTS) especially urgency and postponement, and they needed a longer time to become dry and continent [50].

### ASD and neurology

Mouridsen et al. studied the prevalence of epilepsy among patients with Asperger’s syndrome using the Danish National Hospital Register [51]. In a follow-up period of 4–18 years, patients with Asperger’s syndrome were more likely to have epilepsy (3.9% in 4180 cases) in comparison with the estimated prevalence of 2% in the general population.

### ASD and other medical disorders

Van Tongerloo et al. investigated the presence of characteristic complaints of children with ASD presenting to the general practitioners’ practice, in order to enhance early detection and reduce the delay in diagnosing ASD. This study found that children with ASD presented with more traumata (luxations and distortions) (OR 6.57, *p*-value < 0.01) compared to controls. Also, they were more likely referred to the physiotherapist/ergotherapist (OR 12.63, *p*-value < 0.05), speech therapist (OR 7.07, *p*-value < 0.05) and ear–nose–throat specialist (OR 5.54, *p*-value < 0.05) [52].

Using data from the Swedish Patient Register, Butwicka et al. studied the prevalence of ASD in men with hypospadias (*n* = 9626) [53]. Hypospadias is associated with autism (OR 3.20; 95% CI 2.8–3.8) compared to full brothers (*n* = 4936) and the control group (*n* = 463,100).

In Table 2, the papers are listed that studied ADHD and medical disorders: 12 papers on immunology, 1 paper on neurology and 4 papers on other medical disorders (see Table 2).

### ADHD and immunology

This review includes 12 papers on the association between ADHD and immunological disorders. The study of Leslie et al. [48] showed that prior streptococcal infection was associated with obsessive–compulsive disorder, tic disorders, major depressive disorder. Studies have suggested a link between Group A beta-hemolytic streptococcal (GABHS) infections and the onset or worsening pediatric obsessive–compulsive disorder (OCD), Tourette’s syndrome (TS) and tic disorder. The prepubertal onset of OCD, TS, or tic disorder with abrupt symptom exacerbation after streptococcal infection has been termed PANDAS (pediatric autoimmune neuropsychiatric disorders associated with streptococcal infection). Furthermore, this study showed that some patients prior to their initial diagnosis of ADHD were more likely to have had a diagnosis of streptococcal infection in the previous year than controls (OR 1.20, 95% CI 1.06–1.35) [54]. Although ADHD is frequently comorbid with TS, tic disorders, and OCD, comorbidity of ADHD with TS, tic disorders and OCD were excluded. It remains unclear whether this is the result of a nonspecific stress response or secondary to an activation of the immune system.

The study of Sanchez et al. indicated that frequency of anti-basal ganglia antibodies (ABGA) in children with ADHD (non-comorbid with obsessive–compulsive disorder or tics) does not differ from that in matched controls (4 vs 4%), despite the fact that our ADHD patients had had more recent pharyngeal group A beta-hemolytic streptococcus GABHS infections than the controls (51 vs 14%, *p*-value = 0.007) [55].

With regard to atopy, the study of Schmitt et al. found a prevalence of ADHD among patients with atopic eczema (AE) of 5.2 and 3.4% among controls. Allergic comorbidities (asthma, allergic rhinitis) were not significantly associated with ADHD [56].

The study of Suwan et al. showed significantly increased rates of allergic sensitization (*p*-value = 0.0048) and allergic rhinitis in children with ADHD (*p*-value = 0.008) [57]. On the other hand, no differences were observed regarding other allergic diseases, asthma, eczema, allergic conjunctivitis, food allergy and urticarial (*p*-value > 0.05).

In a population-based study of Tsai et al. allergic rhinitis patients were more likely to have ADHD than the general population (*p*-value < 0.001) [58].

The association between allergy and ADHD was also found by Shyu et al. In their study, allergic children have a higher risk for developing ADHD in comparison with the general population (*p*-value < 0.001) [59]. Allergic rhinitis was found to be the most important contributing factor for the development of ADHD compared to the impact of bronchial asthma and atopic dermatitis.

Chen HJ et al. found a significant association between previously diagnosed diabetes mellitus type 2 and ADHD in children aged 5–15 years (OR 2.75, 95% CI = 1.82–4.16) [60]. There was no association between diabetes type 1 and ADHD.

Using the National Health Insurance Research Database, Chen MH et al. showed that patients with dual diagnoses of ADHD and tic disorder had a significantly higher prevalence of allergic diseases including allergic rhinitis, asthma, atopic dermatitis, allergic conjunctivitis than the ADHD alone group, the tic alone group, and the control group (allergic rhinitis 43 vs. 28.4 vs. 33.6 vs. 19.7%, *p*-value < 0.001, asthma (27.5 vs. 17.2 vs. 18.2 vs. 11.9%, *p*-value < 0.001, atopic dermatitis 10.6 vs. 8.4 vs. 7.0 vs. 5.9%, *p*-value < 0.001, allergic conjunctivitis 55.6 vs. 34.7 vs. 43.5 vs. 26.3%, *p*-value < 0.001) [61].

The study of Tsai et al. found a higher rate of allergic disease in children with ADHD compared to controls, particularly allergic rhinitis (OR 1.80 95% CI 1.69–1.93), and allergic conjunctivitis (OR 1.69; 95% CI 1.58–1.81 [62]. Children with atopic dermatitis (OR 1.80; 95% CI 1.58–2.05) and asthma (OR 1.48; 95% CI 1.24–1.78) were also at higher risk of ADHD. The risk of ADHD increased with numbers of allergic disease and age.

The study of Bekdas et al. presented that patients with ADHD displayed significantly higher positivity for measles IgG (80 vs. 60%, *p*-value = 0.044) [63]. When patients with ADHD were classified according to their pubertal status, adolescents with ADHD displayed higher positivity for mumps.

Chen MH et al. found that the presence of any atopic disease in early childhood increased the risk of developing ADHD (HR: 1.97) in later life [42]. Greater numbers of atopic comorbidities (4 comorbidities HR: 2.53) were significantly related to a greater risk of developing ADHD.

### ADHD and neurology

Using data from the Neurodevelopmental Genomics Cohort Study, Merikangas et al. showed that children with central nervous system conditions (seizures and epilepsy) were significantly more likely to have ADHD (OR 1.30, *p*-value < 0.001) [28]. There was no significant association between ADHD and gastroenterological, immunological, autoimmune, cardiology, hematology, nephrology, endocrine disorders.

### ADHD and other medical disorders

The study of DeMaso et al. found that adolescents with d-transposition of the great arteries showed increased rates of attention deficit/hyperactivity disorder compared to healthy controls (19 vs. 7%, *p*-value = 0.03) [64]. In the study of Dillon et al. 27.8% of the children that underwent adenotonsillectomy had ADHD, whereas only 7.4% of the control group had ADHD [65]. Among the 22 children diagnosed with ADHD before surgery, 50% no longer met diagnostic criteria after adenotonsillectomy.

The population-based study of Silva et al. showed that children under 4 years with ADHD were 70% more likely to be admitted to a hospital compared with controls [66]. Admissions for head injuries, burns, poisons, all other injuries, diseases of the tonsils and adenoids, asthma and early infections were all more common in children with ADHD.

Next to ASD, Butwicka et al. also studied the prevalence of ADHD in men with hypospadias, and found an increased risk of ADHD in men with hypospadias (OR 1.50; 95% CI 1.3–1.9) [53].

## Discussion

The main finding of this systematic review is that medical disorders in children with ASD and ADHD appear to be widespread, e.g., can manifest across different medical areas, such as immunology, neurology and gastroenterology. Although it was not possible to extract prevalence data for the medical disorders, a reasonable number of studies could be included in this review, which supports the notion that children and adolescents with developmental disorders such as ADHD and ASD often suffer from medical disorders that needs to be investigated and addressed [67]. Likewise, children with medical disorders are at increased risk for developmental disorders, which are unfortunately not often recognized [52, 68]. Nevertheless, it is clear that these children require multidisciplinary medical services, including psychiatric help [28].

Increased awareness about the prevalence and types of comorbidity of medical disorders in developmental disorders is important for several reasons. It is first of all relevant for treatment and care for patients and their parents. Also, it may provide important information for fundamental research and could help to create a better understanding of the disease etiology of ASD and ADHD. Furthermore, insight in specific patterns of comorbidity may have important implications for the development of effective interventions for these disorders [5].

However, most of the studies that were identified by our initial search did not reach the required level of quality to come to more profound interpretations, because they were hampered by limitations in study design, in limited case–control matching and/or relied on parent, teacher or self-report of symptoms rather than systematic diagnostic assessments by experts.

First, most studies provide cross-sectional data thus limiting assessment of temporality and inference of causal pathways for the various conditions studied. Second, most studies examine the impact of a single medical comorbid condition, and studies that looked at a broad range of comorbid medical conditions are scarce. Third, the included studies sometimes show inconsistent results.

There are also limitations related to the design of this systematic review. First of all, given the amount of research in child psychiatry, it has been decided to limit the current systematic review to the two main developmental disorders: ASD and ADHD. Consequently, other psychiatric disorders, such as Gilles de la Tourette syndrome or tic disorders were not included in this review. As for sleeping disorder, it was decided to exclude these from the review since sleeping disorders are frequently not viewed as specific medical disorder, but rather as part of a symptom complex of several neuropsychiatric disorders. For a recent review on sleeping disorders in children with neurodevelopmental disorders, see the study by Blackmer and colleagues [69].

Second, strict criteria for methodologic quality of the studies were used for the inclusion and consequently only 29 studies were selected, out of more than 4000 studies that were identified in our initial search. Third, we excluded studies that were not published in English, which might have led to the exclusion of potentially relevant studies.

Bearing these limitations in mind, several strong aspects about this systematic review allow us to conclude that a valuable overview of data is provided. First, the methodologic quality and strength of evidence of the various studies has been assessed in a systematic manner by using a standardized set of criteria. This helps to appreciate individual studies in a correct manner. Second, two raters independently assessed the quality of the studies, selected from two databases, with a standardized set of criteria. An additional rater was consulted in case of a difference in scoring by the other two raters. Third, by using a predefined search strategy, the potential for bias was reduced.

In conclusion, medical comorbidity in children and adolescents with ASD and ADHD appears to occur in numerous medical areas [27, 70]. This should lead to a critical view on current health care systems, which are often marked by clear divisions between medical disciplines. The data of this review clearly point to a multidisciplinary and integrated approach for children and adolescents with ASD and ADHD. Moreover, collaborative care models should be recommended including both a psychiatric and medical approach; that is treatment models including screening for ADHD and ASD in primary care settings, and screening for common medical conditions in child and adolescent psychiatry. Thereby, clinicians should be aware that problem behavior in developmental disorders may be the only symptom of the underlying somatic disorder. Furthermore, prevention programs are needed to address common risk factors for comorbid conditions, such as obesity, underweight, vitamin deficiency, hypo or hyperthyroidism and dyslipidemia. Identifying and treating somatic and psychiatric comorbidities will improve behavior and overall improve quality of life for both patient and family.

Also, developmental disorders such as autism can be a feature of several underlying genetic syndromes, many of which also entail somatic symptoms. As such, a careful analysis of the combination of ASD and somatic disorders requires an extensive diagnostic scheme to detect such genetic syndromes to consider referral to a clinical geneticist. For a review on this specific topic, see the study by Cohen and colleagues [71].

With respect to research in ASD and ADHD, future studies should not only focus on psychiatric symptoms, but provide a broader evaluation of medical disorders, preferably with longitudinal studies. Likewise, it would also be worth to study a specific group of medical disorders and comorbid ADHD and/or ASD. This may ultimately help to provide a more personalized treatment approach and broaden our insight in etiological aspects of ASD and ADHD.
